# A SNP in the 5′ flanking region of the myostatin-1b gene is associated with harvest traits in Atlantic salmon (Salmo salar)

**DOI:** 10.1186/1471-2156-14-112

**Published:** 2013-11-27

**Authors:** Carolina Peñaloza, Alastair Hamilton, Derrick R Guy, Stephen C Bishop, Ross D Houston

**Affiliations:** 1 Division of Genetics and Genomics, The Roslin Institute and Royal (Dick) School of Veterinary Studies, University of Edinburgh, Edinburgh, Midlothian EH25 9RG, UK; 2 Landcatch Natural Selection Ltd, 15 Beta Centre, Stirling University Innovation Park, Stirling FK9 4NF, UK

**Keywords:** Association analysis, Atlantic salmon, Myostatin, Aquaculture, Marker-assisted selection

## Abstract

**Background:**

Myostatin (MSTN) belongs to the transforming growth factor-β superfamily and is a potent negative regulator of skeletal muscle development and growth in mammals. Most teleost fish possess two *MSTN* paralogues. However, as a consequence of a recent whole genome-duplication event, salmonids have four: *MSTN-1* (−*1a* and -*1b*) and *MSTN-2* (−*2a* and -*2b*). Evidence suggests that teleost MSTN plays a role in the regulation of muscle growth. In the current study, the *MSTN-1b* gene was re-sequenced and screened for SNP markers in a commercial population of Atlantic salmon. After genotyping 4,800 progeny for the discovered SNPs, we investigated their association with eight harvest traits - four body-weight traits, two ratios of weight traits, flesh colour and fat percentage - using a mixed model association analysis.

**Results:**

Three novel SNPs were discovered in the *MSTN-1b* gene of Atlantic salmon. One of the SNPs, located within the 5′ flanking region (*g.1086C > T*), had a significant association with harvest traits (p < 0.05), specifically for: Harvest Weight (kg), Gutted Weight (kg), Deheaded Weight (kg) and Fillet Weight (kg). The haplotype-based association analysis was consistent with this result because the two haplotypes that showed a significant association with body-weight traits, hap4 and hap5 (p < 0.05 and p < 0.01, respectively), differ by a single substitution at the *g.1086C > T* locus. The alleles at *g.1086C > T* act in an additive manner and explain a small percentage of the genetic variation of these phenotypes.

**Conclusions:**

The association analysis revealed that *g.1086C > T* had a significant association with all body-weight traits under study. Although the SNP explains a small percentage of the variance, our results indicate that a variation in the 5′ flanking region of the *myostatin* gene is associated with the genetic regulation of growth in Atlantic salmon.

## Background

Myostatin (MSTN) is a potent negative regulator of skeletal muscle development and growth in mammals [1]. Therefore, it has been an attractive candidate gene for the identification of genetic markers for growth and carcass traits in livestock species. Several mutations leading to non-functional MSTN products have been reported to cause the “double-muscling” phenotype characteristic of Piedmontese and Belgian Blue cattle [2,3]. Additionally, a single nucleotide polymorphism (SNP) in the ovine *myostatin* gene has been shown to contribute to the muscular hypertrophy of Texel sheep [4]. Selective breeding for enhanced muscle growth has indirectly increased the frequency of these naturally occurring mutations in meat-producing breeds. Moreover, as a consequence of intense selection double-muscled Belgian Blue cattle are virtually homozygous for the *nt821(del11)* loss-of-function mutation [5]. The *MSTN* gene has also been sequenced in lower vertebrates and invertebrates, such as the zhikong scallop, the bighead carp, the Asian sea bass, the shrimp and amphioxus [6-10]. However, for the majority of non-mammalian species, the functional role of MSTN is poorly defined.

The phylogenetic analysis of *MSTN* genes in teleost fish suggested that a whole genome duplication event, which occurred 320-350 million years ago during early fish radiation [11,12], resulted in two *MSTN* paralogues being present in modern bony fishes (*MSTN-1* and *MSTN-*2). Additionally, in salmonids, each paralogue was duplicated once again likely due to tetraploidization (25-100 million years ago) [13,14]. Thus, a total of four *MSTN* genes can be found in rainbow trout and Atlantic salmon, namely *MSTN-1*(−*1a* and -*1b*) and *MSTN-2*(−*2a* and -*2b*) paralogues. *MSTN* gene structure among teleosts is highly conserved, comprising three exons of comparable size (300–400 nucleotides) separated by two introns, similar to mammals [15]. However, in contrast to mammals, where the expression of a single *MSTN* gene is limited primarily to skeletal muscle [16], teleost fish exhibit a broad pattern of differentially expressed *MSTN* paralogues. For example, *MSTN-1* transcripts have been detected in eye, spleen, muscle and brain, among other tissues [17]. In comparison, *MSTN-2* expression pattern has been shown to be more limited and occurs mostly in the brain [18,19]. While it is unclear exactly what functional role MSTN plays in these tissues, evidence indicates that teleost *MSTN* might be involved in the regulation of muscle development and growth (however, see [15]). In transgenic zebrafish the suppression of the *MSTN* gene by RNA interference has led to a double-muscled phenotype [20], suggesting a similar biological function to that previously described in mammals. This functionality is supported by an increase in the number of muscle fibers (hyperplasia) observed in transgenic Medaka that expresses a dominant-negative *MSTN*[21]. Transgenic trout overexpressing follistatin, one of the more efficacious antagonists of MSTN, also showed enhanced muscle growth due to hyperplasia [22].

The Atlantic salmon (*Salmo salar* L.) is the most important aquaculture species in several countries, including Chile and Norway [23]. Currently, selective breeding programs are being carried out in Norway, Canada, Iceland, Chile and the UK [24]. An important goal of finfish aquaculture is to enhance skeletal muscle growth and improve fillet trait characteristics. Since the Atlantic salmon *MSTN-1b* promoter region harbours *cis*-regulatory elements (E-boxes) that have been shown to bind *in vitro* to the myogenic differentiation factor MyoD [18], a regulatory factor of importance for initiating the myogenic program, it is reasonable to suggest that this paralogue is a strong candidate for regulation of skeletal muscle growth. If true, variation within Atlantic salmon *MSTN-1b* gene may be contributing to the phenotypic variation of economically important harvest traits. Accordingly, relevant polymorphisms could then be applied as tools for marker-assisted selection (MAS) in salmon breeding programs. The objective of this study was to identify novel SNP markers on the *SsMSTN-1b* gene and analyse their association with growth, fatness and colour-related traits in a commercial population of Atlantic salmon.

## Results

### SNP identification and genotypes

The Atlantic salmon *MSTN-1b* gene was re-sequenced to screen for polymorphic variation and to assess the association between this variation and harvest traits. The *MSTN*-contig used for primer design was built by the alignment of the *SsMSTN-1b* gene and two contigs of the Atlantic salmon draft genome assembly (see [25]; NCBI assembly GCA_000233375.1), Contig_064406 and Contig_398167. Following amplification and sequencing of PCR products from ten samples of parental salmon, amplicons generated by two primer pairs (*SaMSTNb23* and *SaMSTNb33*) were found to contain two and one SNP(s), respectively (see Table 1). By re-aligning the PCR products containing the SNPs with *SsMSTN-1b*, their position in the gene was established: two were located in the upstream putative promoter region of the gene (*g.1060C > A* and *g.1086C > T*) and one in the third exon (*c.3501C > G*). For *c.3501C > G*, the substitution of alternative alleles was functionally synonymous.

**Table 1:** **Primer design for PCR analysis of *MSTN *** **candidate gene in Atlantic salmon**

**Gene**	**SNP**	**Primer**	**Sequence (5′-3′)**	**PCR product size (bp)**	**T**_**A **_**(°C)**
*MSTN-1b*	***g.1060C > A*/*g.1086C > T***	SaMSTNb23	AAACTGGGCATTCAATGTTCCACCATACCA	567	55
			CTGCAGTGGCTATTGGGAAAGCTCGCTAAT		
	***c.3501C > G***	SaMSTNb33	ACTTGACGTACGAGCCGAGTTCC	571	50
			TTGCCGCAGCCACACCGACAAC		

A total of 4,800 animals were genotyped. Genotypes and frequencies estimated for the three *MSTN* loci are shown in Table 2. The genotype frequencies of *g.1060C > A* and *g.1086C > T* were consistent with HWE expectations. However, *c.3501C > G* was not in HW equilibrium (p < 0.001), showing a slight deficit of heterozygous animals. The correlations between alleles indicate that *g.1060C > A* and *g.1086C > T* were in strong LD (D’ 0.99, r^2^ 0.38). There was evidence of recombination between *g.1086C > T* and *c.3501C > G* (pairwise D’ 0.52 and r^2^ 0.13).

**Table 2:** Genotype and allele frequencies of SNPs

	***g.1060C > A***			***g.1086C > T***			***c.3501C > G***		
**Genotype**	AA	AC	CC	TT	TC	CC	CC	CG	GG
**Frequency**	74	1128	3474	361	1951	2348	3186	1392	102
**Minor allele frequency**	0.14			0.29			0.17		
**Major allele frequency**	0.86			0.71			0.83		

### Trait properties

The population of 4,800 commercial Atlantic salmon had been measured for several traits at harvest (approximately 3 years of age). The phenotypic mean, standard deviations and ranges of the traits used in this study are given in Table 3 along with their heritability. All of the weight traits (HWT, GWT, DHWT and FLWT) showed similar and high heritabilities (~0.5), whereas yield traits (GYLD and FLYLD) exhibited low estimates (h^2^ < 0.05). Fat content and flesh colour heritabilities were moderate, *viz*. 0.17 and 0.29, respectively.

**Table 3:** **Descriptive statistics and estimates of heritability (h**^**2**^**) for harvest weight (HWT), gutted weight (GWT), deheaded weight (DHWT), fillet weight (FLWT), gutted yield (GYLD), fillet yield (FLYLD), fat content (FAT) and fillet colour (COL) of Atlantic salmon**

**Trait**	**Mean**	**SD**	**Minimum**	**Maximum**	**h**^**2**^
HWT (kg)	2.54	0.63	0.45	7.5	0.52 (0.05)
GWT (kg)	2.32	0.57	0.39	6.8	0.53 (0.05)
DHWT (kg)	2.02	0.51	0.32	6.0	0.51 (0.05)
FLWT (kg)	1.68	0.42	0.23	5.2	0.53 (0.05)
GYLD (%)	0.92	0.01	0.66	0.99	0.04 (0.01)
FLYLD (%)	0.66	0.04	0.28	1.02	0.05 (0.01)
FAT	12.15	5.64	1.1	41.7	0.16 (0.02)
COL	28.92	0.76	24	33	0.29 (0.16)

Standard errors for the heritability of each trait are included in parenthesis.

### Association study

To assess the association between SNP genotype and harvest traits, a mixed model analysis was performed in 4,759 successfully genotyped individuals. A significant association was observed between *g.1086C > T* genotypes (p < 0.05) and all weight phenotypes (HWT, GWT, DHWT and FLWT), but not with any of the other traits. This significance was maintained when *g.1086*C > T was fitted either separately or simultaneously in the model with the other SNPs. Genotypes of *g.1060C > A* and *c.3501C > G* were not significantly associated with any trait when fitted either individually or simultaneously in the model.

To assess the size of effect associated with the significant *MSTN-1b* SNP alleles, the predicted trait values for each genotypic class of *g.1086C > T* were calculated. The thymine allele was associated with an increase in each of the weight traits (p < 0.05), with the additive effect of the SNP on these traits ranging from 30 to 50 g (Table 4). Whilst this effect was significant, the percentage of the additive genetic variance explained by *g.1086C > T* in each trait was less than 1%. The dominance effect was trivial and non-significant.

**Table 4:** Genotype means with standard error, additive effect and dominance effects for the SNP that showed a significant trait association

	**SNP**	**Genotype Mean ± SE**^**1**^				
**Trait**	***g.1086C > T***	**TT**	**TC**	**CC**	***a*** **± SE**	***d*** **± SE**
HWT		2.60 ± 0.04	2.55 ± 0.02	2.50 ± 0.02	0.05 ± 0.017*	−0.0002 ± 0.02
GWT		2.37 ± 0.03	2.33 ± 0.02	2.29 ± 0.02	0.04 ± 0.019*	0.003 ± 0.02
DHWT		2.07 ± 0.03	2.04 ± 0.01	2.00 ± 0.01	0.03 ± 0.017*	0.007 ± 0.01
FLWT		1.73 ± 0.02	1.69 ± 0.01	1.66 ± 0.01	0.03 ± 0.014*	−0.007 ± 0.01

^1^Estimate of the effect is expressed in kg.

*p < 0.05.

Haplotypes were constructed for the three SNPs and the association between haplotype and trait was assessed. In the Atlantic salmon population analysed, 6 haplotypes and 13 diplotypes were identified. Haplotypes with a frequency < 0.01 were excluded from further analysis. The most prevalent haplotype, hap5, accounted for 67% of all haplotypes. The most common diplotype (43% of samples) comprised hap5 homozygotes (Table 5). When haplotype combination was fitted in the model, hap4 showed a significant association with the weight traits (p < 0.05), and the association of hap5 with the same traits was highly significant (p < 0.01); the exception was the fillet weight trait (FLWT), for which only hap5 was significant (p-value = 0.031) (Table 6). These two haplotypes differ in a single nucleotide substitution at *g.1086C > T*, supporting the significant association of this SNP with harvest traits. The occurrence of hap5 at a dosage of two copies in the *SsMSTN-1b* gene was related with a decrease in 60 to 110 g in body weight traits compared to zero copies.

**Table 5:** Frequencies of common haplotypes and diplotypes

**Haplotype**	**Frequency**	**Diplotype**	**Frequency**
ATG (hap1)	0.02	hap5/hap5	0.43
ATC (hap2)	0.12	hap4/hap5	0.21
CTC (hap4)	0.14	hap2/hap5	0.17
CCG (hap5)	0.67	hap5/hap6	0.07
CCC (hap6)	0.05	hap2/hap4	0.03
		hap4/hap4	0.02
		hap1/hap5	0.02
		hap2/hap6	0.01

Haplotypes and Diplotypes with frequency < 0.01 were excluded.

**Table 6:** Means with standard errors for haplotypes that show significant associations with biometrical traits

	Haplotype 4* ± SE^**1**^			Haplotype 5** ± SE^**1**^		
**Trait**	**0**	**1**	**2**	**0**	**1**	**2**
HWT	2.50 ± 0.02	2.56 ± 0.02	2.48 ± 0.06	2.60 ± 0.04	2.53 ± 0.02	2.49 ± 0.02
GWT	2.30 ± 0.02	2.34 ± 0.02	2.28 ± 0.05	2.37 ± 0.03	2.32 ± 0.02	2.28 ± 0.02
DHWT	2.01 ± 0.01	2.04 ± 0.02	1.98 ± 0.05	2.08 ± 0.02	2.02 ± 0.01	1.99 ± 0.02
FLWT	1.68 ± 0.01	1.71 ± 0.01	1.67 ± 0.04	1.71 ± 0.02	1.69 ± 0.01	1.65 ± 0.01

^1^Estimate of the effect is expressed in kg.

*p < 0.05.

**p < 0.01.

## Discussion

In this study, the *SsMSTN-1b* gene was re-sequenced and three novel SNPs were detected: *g.1060C > A* and *g.1086C > T*, both in the 5′ flanking region; and *c.3501C > G*, located in the third exon of the gene. The association analysis showed that *g.1086C > T* had a significant association with the weight traits under study (HWT, GWT, DHWT and FLWT), all of which show a high positive phenotypic correlation (r > 0.97). Quantitative trait loci (QTL) associated with flesh colour and growth traits have been described for Atlantic salmon [26-30]. However, *SsMSTN-1b* is linked to markers mapping to chr25 [18], where only [30] identified a QTL for body-weight in ~38 month-old fish. In general, QTL scans for growth traits in Atlantic salmon suggest that body-weight traits are highly polygenic. As many loci of small effect are expected to be co-regulating traits related to the growth of fish, it is possible that previous QTL mapping studies failed to detect the effect observed in the current study due to a lack of statistical power.

For aquacultural species, a significant association between *MSTN* polymorphisms and production traits has been detected in the bighead carp (*Aristichthys nobilis*), the yellow catfish (*Pelteobagrus fulvidraco*), the spotted halibut (*Verasper variegatus*), the common carp (*Cyprinus carpio*), the Atlantic bay scallop (*Argopecten irradians*) and the gilthead seabream (*Sparus aurata*) [7,31-35]. Interestingly, for marine species the majority of polymorphisms that have been described as having an effect on weight traits (including the present study) are located in non-coding regions of the *MSTN* gene. Although this might be a reflection of low frequency of coding genetic variants in marine species [7,8], it may also be a consequence of the incipient stage of aquacultural research and the shorter history of selective breeding for aquaculture species. For example, it is possible that loss or reduction of function mutations in coding regions of *MSTN* have not yet been observed and/or selected to appreciable frequencies in commercial aquaculture species. Despite the observation that mutations in the coding region of the myostatin gene are known to cause an increase in muscle mass in several mammals, non-coding mutations associated with regulatory pathways may also be underlying phenotypic variation. Moreover, “double-muscling” in Texel sheep is associated with mutations in the 3′UTR of the *MSTN g*ene, which create illegitimate miRNA binding sites and reduce the amount of circulating MSTN protein [4]. It remains to be investigated whether studied marine species encode a more stable MSTN protein, and whether the main effects on growth mediated by this gene are associated with gene regulation or gene structure.

The haplotype-based association analysis was consistent with the significant effect detected for g*.1086C > T*; the two haplotypes that showed a significant association with weight traits, hap4 and hap5 (p < 0.05 and p < 0.01, respectively), differed only by a nucleotide substitution at this locus. In accordance with the predicted trait values of the genotypes at *g.1086C > T*, two copies of the haplotype carrying the unfavourable SNP allele, homozygote hap5/hap5, was associated with a decrease in all body weight traits. The difference between the predicted mean of individuals carrying zero or two copies of hap5 varies between 60 g (for FLWT) to 110 g (for HWT).

*Myostatin* is an important target gene for aquaculture research. Some studies have explored the improvement of growth through the suppression of gene activity by over-expressing the MSTN prodomain (Mstnpro). For example, [36] increased growth rates of rainbow trout by immersing juveniles in bath treatments with flatfish MSTN-1pro expressed in *Escherichia coli.* The improvement of body mass by inhibiting the *myostatin* gene has also been achieved in African catfish (*Clarias gariepinus*), goldfish (*Carassius auratus*) and tilapia (*Oreochromis aureus*) larvae by an immersion bath treatment with a soluble form of the Active Type IIb receptor [37]. However, a study by [38] showed that the positive growth responses achieved by juvenile tilapia under MSTN inhibition by immersion were not sustained until market size; after 45 weeks of exposure to flatfish Mstnpro, no significant weight or length differences between control and immersed tilapia groups were observed.

A promising alternative to short or long-term administration of MSTN inhibiting agents is marker-assisted-selection (MAS) exploiting favourable alleles at naturally segregating polymorphisms in commercial populations of fish. However, in contrast to mammals like the Belgian blue cattle, where a natural deletion of 11 base pairs on the third exon increased from 20–25% muscle mass by hyperplasia [3,5], no genetic variants with large impacts on weight phenotypes have been identified in fish [39]. Our results show that genetic variation at *g.1086C > T* has a significant association with growth traits in a commercial population of Atlantic salmon, although the proportion of variance explained by this marker is relatively small (< 1%). Nonetheless, this SNP should be evaluated further to assess its effect in other populations of salmon and to test any possible functional role on the promoter region of the *SsMSTN-1b* gene. In other domestic animals, it is likely that polymorphisms with large effects on growth traits are rare due to larger selection pressures moving favourable alleles towards fixation [40]. However, Atlantic salmon and other aquaculture species are relatively recently domesticated, and polymorphisms of large effect may feasibly still be segregating in commercial populations. Therefore, future studies should aim at evaluating the effect of additional *MSTN* polymorphisms and other candidate genes for growth in Atlantic salmon. These results would not only be of importance in fish breeding, but also may aid insights into the physiology of muscle growth and development in fish.

## Conclusions

The aim of the study was to discover SNPs in the Atlantic salmon *MSTN-1b* gene and to evaluate their association with growth and fillet traits. Three SNPs were found to be segregating in the fish that were sequenced, and these were then tested in a large commercial population. A SNP located in the upstream region of the gene (*g.1086C > T*) was associated with all body weight traits under study (HWT, GWT, DHWT and FLWT). The alleles in the *g.1086C > T* locus acted in an additive matter, with a change from a *CC* to a *TT* genotype associated with an increase of 70 to 100 g depending on the trait. These results add to the evidence that suggests *SsMSTN-1b*, and potentially the orthologous gene in other teleost species, plays a role in muscle development in fish. The combined effect of further QTL mapping and candidate gene studies assessing the association between genotypes and growth traits may further unravel genes of larger effect and lead to an improved understanding of the regulation of muscle growth in fish.

## Methods

### SNP identification

To discover polymorphisms in the Atlantic salmon *MSTN-1b* locus, specific primer pairs (Table 1) were designed to generate overlapping PCR products from a *MSTN*-contig that contained the entire gene (6394 bp) and ~300 bp of flanking sequence at both ends. This contig was built by aligning the complete *SsMSTN-1b* gene [NCBI: Acc. Num. AJ316006.2] with the Atlantic salmon draft genome assembly ([25]; NCBI assembly GCA_000233375.1). For SNP discovery and the optimization of PCR amplifications, a panel of ten parental individuals was randomly chosen from randomly-selected families of a commercial population of Atlantic salmon (sourced from Landcatch Natural Selection, Ormsary, UK). By searching for polymorphisms in a subset of the parental samples we may have failed to detect some SNPs in our population, particularly those with a rare allele frequency (e.g. < 0.05). However, sampling 10 individuals is adequate to give a good probability of finding SNPs with a minor allele frequency above 0.1. Each PCR product was sequenced using an ABI 3730xl at ARK-Genomics (Roslin, UK) with forward and reverse primers to check for consistency of sequences. SNPs were identified by visual inspection of both chromatograms with BioEdit [41].

### Association study

#### A. Animals and traits

This study was based on 4,800 fish comprising 198 commercial families from Landcatch Natural Selection Ltd. Families were created in 1999 by crossing 136 sires and 198 dams. Pedigree information was available for two previous generations for all individuals with phenotypic records. The phenotypic data was collected at the time of harvesting 3-year old fish and included Harvest Weight (kg), Gutted Weight (kg), Deheaded Weight (kg), Fillet Weight (kg), Gutted Yield (%) and Fillet Yield (%). In addition, Fat Percentage and Fillet Colour were recorded. Fat Percentage was estimated as the mean of eight readings along the animal’s body using the Torry Fatmeter (Distell Ltd). Scores for Fillet Colour ranged from 20–34 units of colour (yellow to red) and derived from the visual contrast of the fillet against the industry standard Roche colour chart (see [42] for details of trait collection).

#### B. DNA extraction and genotyping

Total genomic DNA was extracted from adipose fin tissue using a Biosprint DNA kit (QUIAGEN, Crawley, UK) following the manufacturer’s instruction. The genotyping of the discovered SNPs (see *SNP Identification* above) was performed on all 4,800 fish by LGC Genomics Ltd (Herts, U.K.) using a KASP assay. The KASP assay is a competitive allele-specific PCR-based genotyping system that allows high levels of assay robustness and accuracy (see technology details at http://www.kbioscience.co.uk/reagents/KASP.html). Allele-specific primers were designed and utilised by LGC Genomics based on the supplied gene sequences (see Additional file 1).

#### C. Statistical analysis

##### Test for Hardy-Weinberg equilibrium and linkage disequilibrium

Three SNPs segregating in *SsMSTN-1b* were further tested for departure from Hardy-Weinberg equilibrium (HWE) with a χ^2^ goodness of fit test. Linkage Disequilibrium (LD) was estimated for each pair of SNPs, using both the squared correlation, r^2^, and the normalised linkage disequilibrium coefficient, D’. Both statistical analyses were performed using Haploview 4.2 [43].

##### Haplotype reconstruction

Haplotypes were inferred for individuals using PHASE 2.1 [44,45] and used to perform a haplotype-based association analysis.

##### Mixed model association analysis

Descriptive statistics of harvest traits were performed using the SAS software (SAS Inst., Inc., Cary, NC). The heritability values of each phenotype were calculated from the result of the partitioning of variance components (h^2^ = Va/Vp, where Va and Vp are the additive genetic and the phenotypic variance, respectively) obtained by fitting a single-trait animal model and omitting SNP genotype as a fixed effect, using the ASReml package [46]. To evaluate the relationship between the discovered markers and the harvest traits, SNP genotypes and haplotypes were included in the model as fixed effects.

The mixed model was as follows:

$$Y_{\mathrm{ik}}=µ+G_{k}+a_{i}+e_{\mathrm{ik}}$$

where Y_ik_ is vector of one of the traits on the individual i; μ is the overall mean of the trait; G_k_ is the fixed effect of the SNP genotype k (3 classes) or the effect of the haplotype (coded as the number of copies given per each haplotype described in the population: 0, 1 or 2 copies); a_i_ is the additive effect of the i^th^ animal; and e is the residual term. All available pedigree information was included when fitting the model. Statistical significance of the fixed effects was assessed using the Wald *F-statistics* with denominator degrees of freedom from the fitted model.

##### Predicted SNP genotype effects

For the SNP(s) that showed significant association with a harvest trait, differences between the means of each genotypic class and allelic frequencies were used to estimate additive and dominance effects [47]. Standard errors for both effects were calculated from the variance-covariance matrix of the predicted genotype classes along with the standard errors of their differences (SED). The percentage of the additive genetic variance (%Va) explained by the SNP was determined using the standard formula: 2pq(a + d(q–p))^2^]/Va, where p and q are the major and minor allele frequencies of the SNP, a is the additive effect and d is the calculated dominance effect. The additive genetic variance (Va) was taken from the mixed model without fitting genotype.

## Competing interests

The authors declare that they have no competing interests.

## Authors’ contributions

CP carried out the experiments that led to SNP discovery, performed the statistical analysis and drafted the manuscript. SB and RH conceived the study and helped to draft the manuscript. AH and DG contributed to the collection and management of the harvest data, pedigrees and samples. All authors read and approved the final manuscript.

## Supplementary Material

Additional file 1 **KASP assay template sequence.***SsMSTN-1b* sequences indicating the position and alleles of the three SNP markers. Click here for file
